# Supplementary material for: High-fat diet suppresses the positive effect of creatine supplementation on skeletal muscle function by reducing protein expression of IGF-PI3K-AKT-mTOR pathway
Source: PLoS One. 2018 Oct 4;13(10):e0199728. doi: 10.1371/journal.pone.0199728 (PMC6171830; doi:10.1371/journal.pone.0199728)

S11 Fig. Immunoblotting analyzes of protein expression of phosphorylated AKT (p-Akt-Ser473) from gastrocnemius muscle. The protein level of GAPDH shown at the bottom of immunoblot was used to normalize by the protein levels of pAKT shown at the top. Three independent experiments are shown and separated according to the treatment, indicated with a color-coded bar; green represents SD-T group, blue represents SD-T-CrM group, grey represents HF-T group and red represents HF-T-CrM group. n = 3. Significance was considered as p<0.05.


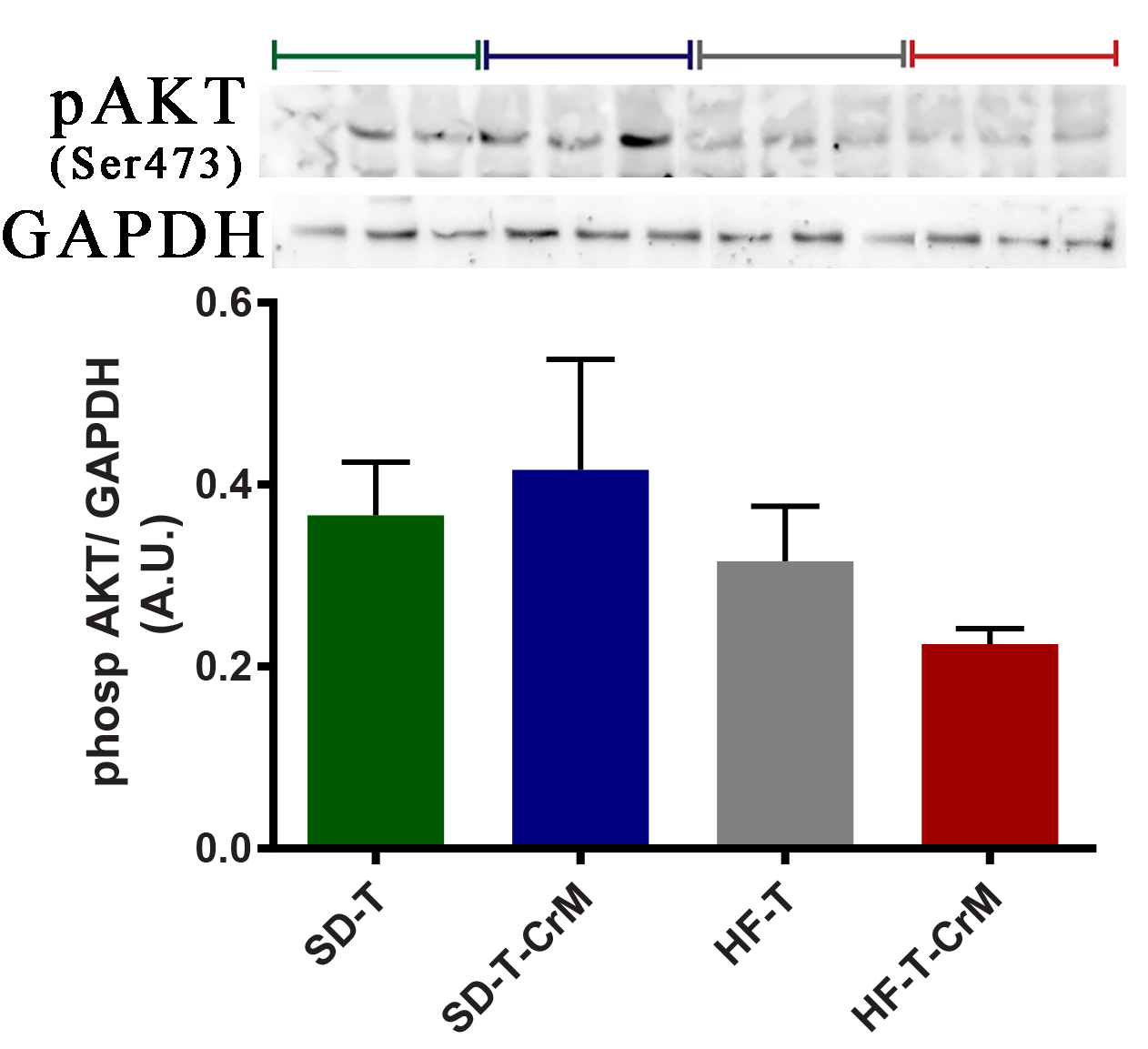

Supplement: S1 Fig — The protein level of GAPDH shown at the bottom of immunoblot was used to normalize by the protein levels of pAKT shown at the top. Three independent experiments are shown and separated according to the treatment, indicated with a color-coded bar; green represents SD-T group, blue represents SD-T-CrM group, grey represents HF-T group and red represents HF-T-CrM group. n = 3. Significance was considered as p<0.05. (DOCX) [file pone.0199728.s001.docx]
