# Supplementary material for: High-fat diet suppresses the positive effect of creatine supplementation on skeletal muscle function by reducing protein expression of IGF-PI3K-AKT-mTOR pathway
Source: PLoS One. 2018 Oct 4;13(10):e0199728. doi: 10.1371/journal.pone.0199728 (PMC6171830; doi:10.1371/journal.pone.0199728)
Supplement: S1 Table — (DOCX) [file pone.0199728.s002.docx]

| **Antibodies** | **Company** | **Catalog Number** | **Dilution** | **Host** | **Clonality** |
| --- | --- | --- | --- | --- | --- |
| GAPDH | Santa Cruz | sc-25778 | 1-1000 | Rabbit | P |
| IGF1 | Santa Cruz | sc-1422 | 1-500 | Goat | P |
| IGF1Rα | Santa Cruz | sc-712 | 1-500 | Rabbit | P |
| IGF1Rβ | Santa Cruz | sc-398250 | 1-500 | Mouse | M |
| IRS1α | Santa Cruz | sc-710 | 1-500 | Rabbit | P |
| IRS1β | Santa Cruz | sc-57342 | 1-500 | Mouse | M |
| p-Akt-Ser473 | Millipore | 05-1003 | 1-1000 | Mouse | M |
| p-mTOR-  Ser2448 | Millipore | 04-385 | 1-1000 | Rabbit | M |
| p70 S6 Kinase | Cell Signaling Technology | 2708 | 1-1000 | Rabbit | P |
| PI3K | Millipore | 09-482 | 1-1000 | Rabbit | P |
| total mTOR | Cell Signaling Technology | 2972 | 1-1000 | Rabbit | P |
| total AKT | Santa Cruz | sc-8312 | 1-1000 | Rabbit | P |
| Anti-rabbit | Sigma-Aldrich | A0545 | 1-4000 | Goat | P |
| Anti-mouse | Sigma-Aldrich | A9044 | 1-4000 | Rabbit | P |
| Anti-goat | Sigma-Aldrich | A5420 | 1-4000 | Rabbit | P |

S1 Table. List of antibodies used on immunoblotting.
