# Supplementary material for: High-fat diet suppresses the positive effect of creatine supplementation on skeletal muscle function by reducing protein expression of IGF-PI3K-AKT-mTOR pathway
Source: PLoS One. 2018 Oct 4;13(10):e0199728. doi: 10.1371/journal.pone.0199728 (PMC6171830; doi:10.1371/journal.pone.0199728)
Supplement: S2 Table — (DOCX) [file pone.0199728.s003.docx]

S2 Table. Comparison of the effect of standard diet (SD) and high-fat diet (HF) on body weight (g) at the end of the 8^th^ week of experiment.

| **Diet** | **SD** | | | **HF** | | |  |
| --- | --- | --- | --- | --- | --- | --- | --- |
| **Treatment** | Mean | SD | n | Mean | SD | n | p |
| **UT** | 509.64 | 29.70 | 5 | 633.32 | 23.94 | 5 | 0.005 |
| **T** | 499.04 | 48.78 | 5 | 695.9 | 61.35 | 5 | <0.0001 |
| **CrM** | 526.6 | 39.86 | 5 | 639.8 | 17.91 | 5 | 0.011 |
| **T-CrM** | 492.44 | 23.09 | 5 | 699.26 | 119.97 | 5 | <0.0001 |
