# Supplementary material for: High-fat diet suppresses the positive effect of creatine supplementation on skeletal muscle function by reducing protein expression of IGF-PI3K-AKT-mTOR pathway
Source: PLoS One. 2018 Oct 4;13(10):e0199728. doi: 10.1371/journal.pone.0199728 (PMC6171830; doi:10.1371/journal.pone.0199728)
Supplement: S3 Table — (DOCX) [file pone.0199728.s004.docx]

S3 Table. Comparison of the effect of standard diet (SD) and high-fat diet (HF) on epididymal fat mass (g) at the end of the 8^th^ week of experiment.

| **Diet** | **SD** | | | **HF** | | |  |
| --- | --- | --- | --- | --- | --- | --- | --- |
| **Treatment** | Mean | SD | n | Mean | SD | n | p |
| **UT** | 9.88 | 1.36 | 5 | 20.02 | 1.41 | 5 | <0.0001 |
| **T** | 9.96 | 3.36 | 5 | 21.78 | 4.22 | 5 | <0.0001 |
| **CrM** | 10.5 | 2.40 | 5 | 24.34 | 2.76 | 5 | <0.0001 |
| **T-CrM** | 10.04 | 3.00 | 5 | 22.7 | 3.24 | 5 | <0.0001 |
