# Supplementary material for: High-fat diet suppresses the positive effect of creatine supplementation on skeletal muscle function by reducing protein expression of IGF-PI3K-AKT-mTOR pathway
Source: PLoS One. 2018 Oct 4;13(10):e0199728. doi: 10.1371/journal.pone.0199728 (PMC6171830; doi:10.1371/journal.pone.0199728)
Supplement: S4 Table — (DOCX) [file pone.0199728.s005.docx]

S4 Table. Comparison of the effect of standard diet (SD) and high-fat diet (HF) on relative fat mass (g) was expressed relative to total body weight (g) at the end of the 8^th^ week of experiment.

| **Diet** | **SD** | | | **HF** | | |  |
| --- | --- | --- | --- | --- | --- | --- | --- |
| **Treatment** | Mean | SD | n | Mean | SD | n | p |
| **UT** | 0.019 | 0.0019 | 5 | 0.032 | 0.0020 | 5 | <0.05 |
| **T** | 0.020 | 0.0055 | 5 | 0.033 | 0.0067 | 5 | <0.05 |
| **CrM** | 0.020 | 0.0044 | 5 | 0.038 | 0.0038 | 5 | <0.05 |
| **T-CrM** | 0.020 | 0.0051 | 5 | 0.033 | 0.0040 | 5 | <0.05 |
