# Supplementary material for: High-fat diet suppresses the positive effect of creatine supplementation on skeletal muscle function by reducing protein expression of IGF-PI3K-AKT-mTOR pathway
Source: PLoS One. 2018 Oct 4;13(10):e0199728. doi: 10.1371/journal.pone.0199728 (PMC6171830; doi:10.1371/journal.pone.0199728)
Supplement: S5 Table — The maximal carrying load was calculated from the total amount of load carried to the top of the ladder. (DOCX) [file pone.0199728.s006.docx]

S5 Table. Summary of the statistical analysis for maximal carrying load (g) between SD-T and SD-T-CrM. The maximal carrying load was calculated from the total amount of load carried to the top of the ladder.

| **Treatment** | **SD-T** | | | **SD-T-CrM** | | |  |
| --- | --- | --- | --- | --- | --- | --- | --- |
| **Week** | Mean | SD | n | Mean | SD | n | p |
| **1** | 426.2 | 53.23 | 5 | 443.48 | 50.88 | 5 | >0.05 |
| **2** | 454.44 | 39.27 | 5 | 648.94 | 67.68 | 5 | 0.0031 |
| **3** | 461.66 | 45.24 | 5 | 799.74 | 86.42 | 5 | <0.0001 |
| **4** | 495.12 | 81.03 | 5 | 827.8 | 131.49 | 5 | <0.0001 |
| **5** | 523.12 | 107.69 | 5 | 822.04 | 133.16 | 5 | <0.0001 |
| **6** | 529.86 | 105.73 | 5 | 861.22 | 119.97 | 5 | <0.0001 |
| **7** | 525.86 | 95.43 | 5 | 856.78 | 152.91 | 5 | <0.0001 |
| **8** | 541.76 | 113.20 | 5 | 894.92 | 125.93 | 5 | <0.0001 |
