# Supplementary material for: High-fat diet suppresses the positive effect of creatine supplementation on skeletal muscle function by reducing protein expression of IGF-PI3K-AKT-mTOR pathway
Source: PLoS One. 2018 Oct 4;13(10):e0199728. doi: 10.1371/journal.pone.0199728 (PMC6171830; doi:10.1371/journal.pone.0199728)
Supplement: S6 Table — The value was calculating the sum of body weight plus maximal carrying load times the successful times the animal climbed the ladder. (DOCX) [file pone.0199728.s007.docx]

S6 Table. Summary of the statistical analysis for total isotonic force (g) between SD-T and SD-T-CrM. The value was calculating the sum of body weight plus maximal carrying load times the successful times the animal climbed the ladder.

| **Treatment** | **SD-T** | | | **SD-T-CrM** | | |  |
| --- | --- | --- | --- | --- | --- | --- | --- |
| **Week** | Mean | SD | n | Mean | SD | n | p |
| **1** | 1382.40 | 263.85 | 5 | 1894.52 | 596.52 | 5 | >0.05 |
| **2** | 2405.34 | 511.16 | 5 | 4437.30 | 789.26 | 5 | 0.0013 |
| **3** | 2362.00 | 544.10 | 5 | 4162.99 | 513.93 | 5 | 0.0006 |
| **4** | 2333.76 | 822.56 | 5 | 3678.53 | 446.76 | 5 | 0.0123 |
| **5** | 2699.23 | 507.29 | 5 | 3578.46 | 644.96 | 5 | 0.0434 |
| **6** | 2537.9 | 525.41 | 5 | 3987.80 | 479.56 | 5 | 0.0018 |
| **7** | 2448.51 | 468.67 | 5 | 3570.45 | 867.57 | 5 | 0.0344 |
| **8** | 2479.02 | 727.357 | 5 | 4406.45 | 921.37 | 5 | 0.0063 |
