# Supplementary material for: High-fat diet suppresses the positive effect of creatine supplementation on skeletal muscle function by reducing protein expression of IGF-PI3K-AKT-mTOR pathway
Source: PLoS One. 2018 Oct 4;13(10):e0199728. doi: 10.1371/journal.pone.0199728 (PMC6171830; doi:10.1371/journal.pone.0199728)
Supplement: S7 Table — Work was calculated multiplying total mass lifted to the top of the ladder, the length of the ladder (1.1m), gravitational force (9.8 06 ms-2) and the ladder’s angle (sen80 = 0.9848). (DOCX) [file pone.0199728.s008.docx]

S7 Table. Summary of the statistical analysis for work (kJ) between SD-T and SD-T-CrM. Work was calculated multiplying total mass lifted to the top of the ladder, the length of the ladder (1.1m), gravitational force (9.8 06 ms^-2^) and the ladder’s angle (sen80 = 0.9848).

| **Treatment** | **SD-T** | | | **SD-T-CrM** | | |  |
| --- | --- | --- | --- | --- | --- | --- | --- |
| **Week** | Mean | SD | n | Mean | SD | n | p |
| **1** | 50.39 | 8.67 | 5 | 74.10 | 9.57 | 5 | 0.0034 |
| **2** | 52.42 | 11.78 | 5 | 82.84 | 13.15 | 5 | 0.0049 |
| **3** | 50.90 | 12.33 | 5 | 83.81 | 7.62 | 5 | 0.0010 |
| **4** | 54.28 | 15.85 | 5 | 76.20 | 6.64 | 5 | 0.0213 |
| **5** | 55.93 | 15.22 | 5 | 71.52 | 6.05 | 5 | >0.05 |
| **6** | 56.43 | 13.41 | 5 | 64.23 | 13.98 | 5 | >0.05 |
| **7** | 49.58 | 11.86 | 5 | 64.88 | 13.77 | 5 | >0.05 |
| **8** | 52.59 | 11.12 | 5 | 62.74 | 4.41 | 5 | >0.05 |
