# Supplementary material for: High-fat diet suppresses the positive effect of creatine supplementation on skeletal muscle function by reducing protein expression of IGF-PI3K-AKT-mTOR pathway
Source: PLoS One. 2018 Oct 4;13(10):e0199728. doi: 10.1371/journal.pone.0199728 (PMC6171830; doi:10.1371/journal.pone.0199728)
Supplement: S8 Table — The maximal carrying load was calculated from the total amount of load carried to the top of the ladder. (DOCX) [file pone.0199728.s009.docx]

S8 Table. Summary of the statistical analysis for maximal carrying load (g) between HF-T and HF-T-CrM. The maximal carrying load was calculated from the total amount of load carried to the top of the ladder.

| **Treatment** | **HF-T** | | | **HF-T-CrM** | | |  |
| --- | --- | --- | --- | --- | --- | --- | --- |
| **Week** | Mean | SD | n | Mean | SD | n | p |
| **1** | 437.90 | 95.51 | 5 | 551 | 47.65 | 5 | >0.05 |
| **2** | 589.83 | 137.27 | 5 | 621.1 | 14.06 | 5 | >0.05 |
| **3** | 691.93 | 68.02 | 5 | 733.64 | 63.08 | 5 | >0.05 |
| **4** | 785.43 | 86.51 | 5 | 790.58 | 90.30 | 5 | >0.05 |
| **5** | 847.63 | 90.55 | 5 | 809.44 | 48.03 | 5 | >0.05 |
| **6** | 864.70 | 106.90 | 5 | 790.26 | 118.29 | 5 | >0.05 |
| **7** | 812.08 | 59.81 | 5 | 762.4 | 112.25 | 5 | >0.05 |
| **8** | 814.60 | 124.68 | 5 | 880.4 | 163.047 | 5 | >0.05 |
