# Supplementary material for: High-fat diet suppresses the positive effect of creatine supplementation on skeletal muscle function by reducing protein expression of IGF-PI3K-AKT-mTOR pathway
Source: PLoS One. 2018 Oct 4;13(10):e0199728. doi: 10.1371/journal.pone.0199728 (PMC6171830; doi:10.1371/journal.pone.0199728)
Supplement: S9 Table — The value was calculating the sum of body weight plus maximal carrying load times the successful times the animal climbed the ladder. (DOCX) [file pone.0199728.s010.docx]

S9 Table. Summary of the statistical analysis for total isotonic force (g) between HF-T and

HF-T-CrM. The value was calculating the sum of body weight plus maximal carrying load times the successful times the animal climbed the ladder.

| **Treatment** | **HF-T** | | | **HF-T-CrM** | | |  |
| --- | --- | --- | --- | --- | --- | --- | --- |
| **Week** | Mean | SD | n | Mean | SD | n | p |
| **1** | 1417.79 | 552.80 | 5 | 2311.9 | 473.75 | 5 | >0.05 |
| **2** | 3154.27 | 763.69 | 5 | 4222.31 | 395.043 | 5 | >0.05 |
| **3** | 3490.68 | 464.26 | 5 | 3816.95 | 386.83 | 5 | >0.05 |
| **4** | 3734.49 | 1115.72 | 5 | 3535.11 | 481.34 | 5 | >0.05 |
| **5** | 4357.74 | 671.61 | 5 | 3355.93 | 520.53 | 5 | >0.05 |
| **6** | 4145.93 | 350.57 | 5 | 3653.71 | 420.24 | 5 | >0.05 |
| **7** | 3915.18 | 220.53 | 5 | 3166.15 | 635.28 | 5 | >0.05 |
| **8** | 3528.74 | 653.08 | 5 | 4264.07 | 531.80 | 5 | >0.05 |
