# Supplementary material for: High-fat diet suppresses the positive effect of creatine supplementation on skeletal muscle function by reducing protein expression of IGF-PI3K-AKT-mTOR pathway
Source: PLoS One. 2018 Oct 4;13(10):e0199728. doi: 10.1371/journal.pone.0199728 (PMC6171830; doi:10.1371/journal.pone.0199728)
Supplement: S10 Table — Work was calculated multiplying total mass lifted to the top of the ladder, the length of the ladder (1.1m), gravitational force (9.8 06 ms-2) and the ladder’s angle (sen80° = 0.9848). (DOCX) [file pone.0199728.s011.docx]

S10 Table. Summary of the statistical analysis for work (kJ) between HF-T and HF-T-CrM. Work was calculated multiplying total mass lifted to the top of the ladder, the length of the ladder (1.1m), gravitational force (9.8 06 ms^-2^) and the ladder’s angle (sen80 = 0.9848).

| **Treatment** | **HF-T** | | | **HF-T-CrM** | | |  |
| --- | --- | --- | --- | --- | --- | --- | --- |
| **Week** | Mean | SD | n | Mean | SD | n | p |
| **1** | 49.62 | 12.97 | 5 | 62.59 | 9.19 | 5 | >0.05 |
| **2** | 68.20 | 19.84 | 5 | 62.97 | 14.75 | 5 | >0.05 |
| **3** | 62.18 | 4.80 | 5 | 72.65 | 7.16 | 5 | >0.05 |
| **4** | 53.89 | 4.745 | 5 | 71.54 | 8.31 | 5 | >0.05 |
| **5** | 63.39 | 9.66 | 5 | 69.73 | 6.08 | 5 | >0.05 |
| **6** | 70.16 | 4.28 | 5 | 62.30 | 10.84 | 5 | >0.05 |
| **7** | 62.09 | 4.91 | 5 | 73.19 | 14.58 | 5 | >0.05 |
| **8** | 62.82 | 4.85 | 5 | 75.48 | 10.81 | 5 | >0.05 |
